# Incidence of symptomatic image‐confirmed venous thromboembolism in outpatients managed in a hospital‐led COVID‐19 virtual ward

**DOI:** 10.1002/jha2.305

**Published:** 2021-10-01

**Authors:** Susan Shapiro, Karim Fouad Alber, Joshua Morton, George Wallis, Meriel Britton, Alex Bunn, Hashem Cheema, Saman Jalilzadeh Afshari, Ei Chae Zun Lin, Oliver Madge, Saniya Naseer, Esther Ng, Alexander Pora, Abbas Sardar, Andrew Brent, Daniel Lasserson

**Affiliations:** ^1^ Oxford Haemophilia and Thrombosis Centre Oxford University Hospitals NHS Foundation Trust Oxford UK; ^2^ Radcliffe Department of Medicine University of Oxford Oxford UK; ^3^ NIHR Oxford Biomedical Research Centre Oxford UK; ^4^ Ambulatory Assessment Unit Oxford University Hospitals NHS Foundation Trust Oxford UK; ^5^ Infectious Diseases Department Oxford University Hospitals NHS Foundation Trust Oxford UK; ^6^ Nuffield Department of Medicine University of Oxford Oxford UK; ^7^ Warwick Medical School University of Warwick Coventry UK

**Keywords:** ambulatory, COVID‐19, outpatient, SARS‐CoV2, thromboprophylaxis, thrombosis, venous thromboembolism, virtual

During the course of the coronavirus disease 2019 (COVID‐19) pandemic, there has been a drive for ambulatory medical teams to develop ‘virtual COVID‐19 wards’ to enable safe care at home for people with COVID‐19 and support prioritization of hospital beds for those most in need; and this form of care is likely to continue to expand. Although hospitalized patients with COVID‐19 have high rates of venous thromboembolism (VTE) [1], the VTE rate for ambulatory patients is not known, and COVID‐19 guidelines either do not address VTE prevention in this setting or give conflicting recommendations [2, 3, 4, 5].

Oxford University Hospitals is one of the largest UK teaching hospitals and reviews 14,000 patients annually in the adult medical ambulatory care unit. In November 2020, we initiated a hospital‐led COVID‐19 virtual ward (Covid Care at Home, CC@H). In brief, patients with clinically suspected or confirmed COVID‐19 could be referred to CC@H after assessment by acute medical teams or the Emergency Department, if deemed well enough to go home but at risk of deterioration. Referral criteria included ‘clinician concern’ as well as specific vulnerable groups (e.g. age > 50 years, pre‐existing co‐morbidities). Patients were given an oxygen saturation monitor and received a structured telephone‐call at least daily for 14 days to collect oxygen saturations at rest/on exertion, symptom trajectory and any bleeding concerns. Patients with signs of deterioration had face‐to‐face review arranged. Local expert consensus guidance was to recommend 7 days pharmacological thromboprophylaxis unless the patient was at high bleeding risk (this is in‐line with subsequently published NICE COVID‐19 guidance to consider pharmacological thromboprophylaxis for hospital‐led community care patients unless high bleeding risk) [3]. The local guidance recommended dalteparin 5000 units subcutaneously (dose adjusted for extremes of body weight, < 40 and > 120 kg); and if dalteparin could not be administered, then rivaroxaban 10 mg could be considered. We determined to measure the rate of symptomatic VTE and bleeding at 6 weeks.

Data collection was approved by Oxford University Hospitals (audit number 7014). Consecutive adult patients admitted to CC@H between 16 November 2020 and 19 February 2021 were identified through CC@H records. Data were obtained from electronic patient records from time of presentation to CC@H and for 6 weeks or until known death. Six weeks was chosen as the risk of VTE associated with medical hospitalization is highest for the first 6 weeks [6]. Bleeding events were classified according to ISTH definitions [7, 8]. The data that support the findings of this study are available from the corresponding author upon reasonable request.

Between 16 November 2020 and 19 February 2021, 281 adult patients were referred to CC@H. This included 129 patients with prior hospitalization > 48 h, and 7 asymptomatic dialysis patients with SARS‐CoV2 detected on screening (these patients were accepted by CC@H to support early ward discharge and safety netting). In order to analyse VTE and bleeding events in high‐risk symptomatic COVID‐19 outpatients, patients with prior hospitalization > 48 h and asymptomatic dialysis patients were excluded from further analysis. Of the 145 included patients, 108 patients were successfully contacted for the planned 6‐week follow‐up phone‐call at a median of 59 days (interquartile range, IQR 45–94) after discharge. Patient characteristics are shown in Table 1 and key events within the 6‐week period following acceptance by CC@H are described in Table 2. Patients were not screened for VTE but underwent investigations (CT pulmonary angiogram and whole leg ultrasound Doppler) if clinical suspicion: 37 patients were imaged at initial presentation, and 18 patients following acceptance by the virtual ward [with median time to re‐presentation and imaging of 4 days (IQR 2–10)]. One patient was diagnosed with multiple segmental pulmonary emboli within 36 h of initial referral to CC@H (Padua score 5). One patient (Padua score 1) was diagnosed with a below knee deep vein thrombosis (DVT) at 17 days after CC@H referral and opted for two additional weekly serial scans to exclude extension to proximal DVT as opposed to starting anticoagulation. One patient (0.7%) experienced ‘clinically‐relevant‐non‐major‐bleeding’ and seven (4.8%) reported minor bleeds (Table 2).

**TABLE 1:** Characteristics of outpatients managed under the COVID‐19 virtual ward

Characteristics	Total number (%)
Confirmed COVID‐19^a^	138 (95.2%)
Suspected COVID‐19^b^	7 (4.8%)
No preceding hospitalization before virtual ward referral	114 (78.6%)
Preceding hospitalization ( < 48 h) before virtual ward	31 (21.4%)

Demographics	Median (IQR)
Age (years)	51 (44–63)
Weight (kg)	83.6 (70.0–94.8)
Body mass index (BMI) (kg/m^2^)	31 (26.5–33.8)

Sex and co‐morbidities	Total number (%)
Sex (male)	82 (56.6)
No co‐morbidities	54 (37.2)
Hypertension	41 (28.3)
Diabetes	33 (22.8)
COPD	7 (4.8)
Asthma	25 (17.2)
Ischaemic heart disease	9 (6.2)
Peripheral vascular disease	1 (0.7)
Previous stroke	2 (1.4)
Total venous thromboembolism	9 (6.2)
Previous venous thromboembolism	5 (3.4)
Acute COVID‐19‐related thromboembolism	4 (2.8)
Malignancy	12 (8.3)
Chronic kidney disease	12 (8.3)
Dialysis	4 (2.8)
Obesity	41 (28.3)

Anti‐platelets/anti‐coagulants (before COVID‐19 virtual ward referral)	Total number (%)
Aspirin	12 (8.3)
Clopidogrel	3 (2.1)
Ticagrelor	1 (0.7)
Warfarin	2 (1.4)
Direct oral anticoagulant (DOAC)^c^	11 (7.6)
^c^(DOAC initiated for acute pulmonary embolus < 24 h prior)	4 (2.8)
Observations at initial presentation	Mean (IQR)
Oxygen saturations (%)^d^	95.8 (94–97)
^d^ Number of people on supplementary oxygen 1–5 L	9 (6.5%)
Respiratory rate (breaths per minute)	20.8 (18–22)
Temperature (°C)	37.2 (36.5–38.0)
Heart rate (beats per minute)	94.9 (84–104)

Laboratory values at initial presentation	Median (IQR)
Haemoglobin g/L	139 (129–149)
White cell count x10^9^/L	6.0 (4.4–7.8)
Platelet count x10^9^/L	218 (174–261)
Neutrophils x10^9^/L	3.8 (2.7–5.5)
Lymphocytes x10^9^/L	1.1 (0.8–1.6)
Prothrombin time (s)	10.6 (10.3–10.9)
Activate partial thromboplastin time (s)	23.8 (22.0–25.3)
D‐dimer μg/L	537 (355.5–850.8)
C‐reactive protein (CRP) mg/L	44.6 (20.3–75.4)

^a^Confirmed COVID‐19: positive for SARS‐CoV‐2 by reverse transcriptase PCR on a nose/throat swab.

^b^Suspected COVID‐19: negative for SARS‐CoV‐2 by reverse transcriptase PCR on a nose/throat swab but considered to have had COVID‐19 on independent clinical review.

**TABLE 2:** Thromboprophylaxis regimen and events in outpatients managed under the COVID‐19 virtual ward

Thromboprophylaxis under virtual ward	Total number (%)	Detailed characterization
Dalteparin (weight‐based dosing)	84 (57.9)	
Dalteparin 5000 units daily	82 (56.6)	
Dalteparin 7500 units daily	2 (1.4)	
Rivaroxaban 10 mg daily	42 (29.0)	
None	6 (4.1)	Two patients had documented bleeding risk; four patients reason for not prescribing thromboprophylaxis unknown
Therapeutic anticoagulation	13 (9.0)	Seven for atrial fibrillation, two for secondary VTE prevention and four for acute pulmonary emboli (one lobar and three multiple segmental pulmonary emboli) diagnosed prior to virtual ward referral

Events with 42 days or referral to virtual ward	Total number (%)	
Re‐presentation to hospital	46 (31.7)	Almost all (43 of 46) re‐presentations to hospital were due to COVID‐19‐related reasons with the commonest causes being breathlessness and hypoxia; the remaining three were due to fall, sore throat and nose‐bleed. Median time to re‐attendance was 2 days (IQR 1–5.5) and to admission was 3 days (IQR 2–6). For the 25 people who were subsequently admitted for a deterioration secondary to COVID, the median duration hospitalization was 4.5 days (IQR 3–7.3).
Review only	21 (14.5)	
Subsequent admission	25 (17.2)	
Bleeding	8 (5.5%)	One patient had CRNMB: nose‐bleed resulting in re‐attendance for review and dalteparin prophylaxis being withheld for a day. The site of the reported seven minor bleeds was: four nasal (often after nasal swabs), one oro‐mucosal and two haemorroidal. At time of bleeding events, one patient was receiving therapeutic anticoagulation, six thromboprophylaxis (three dalteparin 5000 units OD, three rivaroxaban 10 mg OD) and none were additionally taking anti‐platelets.
Major bleeding	0 (0.0)	
CRNMB	1 (0.7)	
Minor bleeding	7 (4.8%)	
New diagnosis of VTE	2 (1.4)	One PE diagnosed within 36 h following virtual ward referral (considered to be not preventable by virtual ward thromboprophylaxis and potentially present at initial presentation), treated with anticoagulation. One below knee DVT diagnosed 17 days after initial virtual ward referral, patient opted for weekly serial scanning for additional 2 weeks to exclude extension to proximal DVT instead of starting anticoagulation.
Death	3 (2.1)	Autopsies were not performed. Cause of death was listed as COVID‐19 infection in all patients. The mean age at death was 87 years (range 85–89); all had significant co‐morbidities and frailty. Two patients died in a care home setting and one died in hospital. Median time to death from admission under COVID‐19 virtual ward was 4 days (IQR 3.5–7.5) and median time from onset of symptoms to death was 8 days (IQR 7–11 days).

Padua score	Total number (%)	Padua score for the 132 patients not on anticoagulation at point of referral to virtual ward
<4	114 (86.4%)	Of these 114 patients, 110 (96.5%) were prescribed thromboprophylaxis. The four patients in this group not prescribed thromboprophylaxis did not have a clearly documented reason as to why.
≥4	18 (13.6%)	Of these 18 patients, 16 (88.9%) were prescribed thromboprophylaxis. The two patients in this group not prescribed thromboprophylaxis had documented bleeding risk (thrombocytopenia secondary to haematological malignancy and cirrhosis with associated thrombocytopaenia)

Note: ISTH bleeding definitions [7, 8]: major bleeding (MB) is fatal bleeding, bleeding into a critical organ, bleeding causing more than 20 g/L fall in haemoglobin or transfusion of 2 or more units of red cells; clinically relevant non‐major bleeding (CRNMB) is bleeding that does not meet major criteria but requires medical intervention or hospitalization. Bleeding was recorded as minor if bleeding was reported which did not meet the definition of CRNMB or MB.

Abbreviations: CRNMB, clinically relevant non‐major bleeding; DOAC, direct oral anticoagulant; DVT, deep vein thrombosis; MB, major bleeding; PE, pulmonary embolus; VTE, venous thromboembolism.

In this cohort of 145 consecutive outpatients under a hospital‐led COVID‐19 virtual ward, of whom 13 (9%) had an indication for therapeutic anticoagulation and 126 (86.9%) were prescribed 7 days thromboprophylaxis, two (1.4%) developed a VTE within 42 days. One event was diagnosed within 36 h of initial referral and is considered a delayed diagnosis and not preventable by CC@H initiated thromboprophylaxis, and one was a below knee DVT. This VTE rate is significantly lower than the 7.2% VTE incidence in our inpatients with COVID‐19 [9]. This is noteworthy as the patients referred to CC@H were considered at risk for deteriorating with moderate‐severe COVID‐19, with 62.8% having at least one co‐morbidity, 31.3% re‐attending and 18.1% requiring subsequent hospitalization. Of note, if we had restricted guidelines to consider thromboprophylaxis (in those not already on anticoagulation) to patients with Padua VTE risk assessment [10] ≥4, then only 17.4% would have been eligible.

A retrospective study of 30‐day incidence of VTE in 220,588 adults tested for COVID‐19 did not find a significant increase in outpatient VTEs in those who had positive tests compared to those with negative tests (1.8% vs. 2.2%, *p* = 0.16) [11]. While this suggests the risk of VTE is generally low in COVID‐19 outpatients, the population was very heterogeneous, including for COVID‐19 severity. Several randomized controlled trials (RCTs) are underway to study enoxaparin, apxiaban, rivaroxaban and aspirin in outpatients with COVID‐19 (e.g. NCT04498273, NCT04492254 and NCT04400799) [12], and reviewing the results in conjunction with co‐morbidities and severity of COVID‐19 may help inform VTE prevention strategies for symptomatic patients at risk of deterioration in hospital‐led virtual wards in the future.

The strengths of our single‐centre observational study are the structured data collected during telephone‐calls for the initial 14 days and 6‐week follow‐up. Limitations include the relatively small number of patients. Events at 42 days may be under‐reported in the 26% of patients who we were unable to contact for 6‐week telephone‐call; however, the risk is considered to be low because the majority are a local population who would re‐present to Oxford with complications, and additionally, we have an established network with surrounding hospitals for informing us of post‐discharge VTEs as part of the national VTE prevention programme [13, 14].

In summary, this report highlights the lack of data to guide VTE prevention strategies for hospital‐led COVID‐19 virtual wards. A strategy of 7 days thromboprophylaxis was associated with relatively low VTE rates and no major bleeding in this cohort of patients considered at risk of deterioration. It highlights the need for further data, including future prospective RCTs of VTE prevention in this expanding setting of hospital‐led ambulatory care.

## CONFLICTS OF INTEREST

SS has received educational speaker fees from Bayer and Pfizer, conference support from Bayer and advisory board fees from Pfizer. The other authors have no potential conflicts of interest to declare.

## AUTHOR CONTRIBUTIONS

SS and DL conceived and designed the study. KFA, JM, GW, MB, AB, HC, SJA, ECZL, OM, SN, EN, AP and AS collected data. KFA, SS, AB and DL analysed data. SS drafted the manuscript. All authors critically revised the manuscript and approved the final version.
